# Semantical Analysis of Contextual Types

**DOI:** 10.1007/978-3-030-45231-5_26

**Published:** 2020-04-17

**Authors:** Brigitte Pientka, Ulrich Schöpp

**Affiliations:** 8 ENS/CNRS, Université Paris-Saclay, Cachan, France; 9 University of Duisburg-Essen, Duisburg, Germany; 10 McGill University, Montreal, Canada; 11 fortiss GmbH, Munich, Germany

## Abstract

We describe a category-theoretic semantics for a simply typed variant of Cocon, a contextual modal type theory where the box modality mediates between the weak function space that is used to represent higher-order abstract syntax (HOAS) trees and the strong function space that describes (recursive) computations about them. What makes Cocon different from standard type theories is the presence of first-class contexts and contextual objects to describe syntax trees that are closed with respect to a given context of assumptions. Following M. Hofmann’s work, we use a presheaf model to characterise HOAS trees. Surprisingly, this model already provides the necessary structure to also model Cocon. In particular, we can capture the contextual objects of Cocon using a comonad $\flat$ that restricts presheaves to their closed elements. This gives a simple semantic characterisation of the invariants of contextual types (e.g. substitution invariance) and identifies Cocon as a type-theoretic syntax of presheaf models. We express our category-theoretic constructions by using a modal internal type theory that is implemented in Agda-Flat.

### 1 Introduction

A fundamental question when defining, implementing, and working with languages and logics is: How do we represent and analyse syntactic structures? Higher-order abstract syntax [19] (or lambda-tree syntax [17]) provides a deceptively simple answer to this question. The basic idea to represent syntactic structures is to map uniformly binding structures in our object language (OL) to the function space in a meta-language thereby inheriting $\alpha$-renaming and capture-avoiding substitution. In the logical framework LF [10], for example, we can define a small functional programming language consisting of functions, function application, and let-expressions using a type tm as follows:

```
lam : (tm → tm) → tm.      letv: tm → (tm → tm) → tm.
app : tm → tm → tm.
```

The object-language term $(\mathsf{lam}\ x.\,\mathsf{lam}\ y.\,\mathsf{let}\ w = x\ y\ \mathsf{in}\ w\ y)$ is then encoded as `lam λx.lam λy.letv (app x y) λw.app w y` using the LF abstractions to model binding. Object-level substitution is modelled through LF application; for instance, the fact that $((\mathsf{lam}\ x.M)\ N)$ reduces to $[N/x]M$ in our object language is expressed as `(app (lam M) N)` reducing to `(M N)`.

This approach is elegant and can offer substantial benefits: we can treat objects equivalent modulo renaming and do not need to define object-level substitution.

However, we not only want to just construct HOAS trees, but also to analyse them and to select sub-trees. This is challenging, as sub-trees are context sensitive. For example, the term `letv (app x y)` $\lambda$`w.app w y` only makes sense in a context `x:tm,y:tm`. Moreover, one cannot simply extend LF to allow syntax analysis. If one simply added a recursion combinator to LF, then it could be used to define many functions `M: tm` $\to$ `tm` for which `lam M` would not represent an object-level syntax term [12].

Contextual types [18,20] offer a type-theoretic solution to these problems by reifying the typing judgement, i.e. that `letv (app x y)` $\lambda$`w.app w y` has type `tm` in the context `x:tm,y:tm`, as a *contextual type* $\lceil x{:}\mathtt{tm}, y{:}\mathtt{tm} \vdash \mathtt{tm}\rceil$. The contextual type $\lceil x{:}\mathtt{tm}, y{:}\mathtt{tm} \vdash \mathtt{tm}\rceil$ describes a set of terms of type `tm` that may contain variables $x$ and $y$. In particular, the contextual object $\lceil x, y \vdash \mathtt{letv}\ (\mathtt{app}\ x\ y)\ \lambda w.\mathtt{app}\ w\ y\rceil$ has the given contextual type. By abstracting over contexts and treating contexts as first-class, we can now recursively analyse HOAS trees [20,25,21]. Recently, [23] further generalised these ideas and presented a contextual modal type theory, Cocon, where we can mix HOAS trees and computations, i.e. we can use (recursive) computations to analyse and traverse (contextual) HOAS trees and we can embed computations within HOAS trees. This line of work provides a syntactic perspective to the question of how to represent and analyse syntactic structures with binders, as it focuses on decidability of type checking and normalisation. However, its semantics remains not well-understood. What is the semantic meaning of a contextual type? Can we semantically justify the given induction principles? What is the semantics of a first-class context?

While a number of closely related categorical models of abstract syntax with bindings [12,8,9] were proposed around 2000, the relationship of these models to concrete type-theoretic languages for computing with HOAS structures was teneous. In this paper, we give a category-theoretic semantics for Cocon (for simply-typed HOAS). This provides semantic perspective of contextual types and first-class contexts. Maybe surprisingly, the presheaf model introduced by Hofmann [12] already provides the necessary structure to also model contextual modal type theory. Besides the standard structure of this model, we only need two additional concepts: a $\flat$-modality and a cartesian closed universe of representables. For simplicity and lack of space, we focus on the special case of Cocon where the HOAS trees are simply-typed. Concentrating on the simply-typed setting allows us to introduce the main idea without the additional complexity that type dependencies bring with them. We outline the dependently-typed case in Sec. 6.

Our work provides a semantic foundation to Cocon and can serve as a starting point to investigate connections to similar work. First, our work connects Cocon to other work on internal languages for presheaf categories with a $\flat$-modality, such as spatial type theory [27] or crisp type theory [16]. Second, it may help to understand the relations of Cocon to type theories that use a modality for metaprogramming and intensional recursion, such as [15]. While Cocon is built on the same general ideas, a main difference seems to be that Cocon distinguishes between HOAS trees and computations, even though it allows mixed use of them. We hope to clarify the relation by providing a semantical perspective.

### 2 Presheaves for Higher-Order Abstract Syntax

Our work begins with the presheaf models for HOAS of [12,8]. The key idea of those approaches is to integrate substitution-invariance in the computational universe in a controlled way. For the representation of abstract syntax, one wants to allow only substitution-invariant constructions. For example, `lam M` represents an object-level abstraction if and only if `M` is a function that uses its argument in a substitution-invariant way. For computation with abstract syntax, on the other hand, one wants to allow non-substitution-invariant constructions too. Presheaf categories allow one to choose the desired amount of substitution-invariance.

Let $\mathbb{D}$ be a small category. The presheaf category $\widehat{\mathbb{D}}$ is defined to be the category $\mathrm{Set}^{\mathbb{D}^{\mathrm{op}}}$. Its objects are functors $F\colon \mathbb{D}^{\mathrm{op}} \to \mathrm{Set}$, which are also called *presheaves.* Such a functor $F$ is given by a set $F(\Psi)$ for each object $\Psi$ of $\mathbb{D}$ together with a function $F(\sigma)\colon F(\Phi) \to F(\Psi)$ for any object $\Phi$ and $\sigma\colon \Psi \to \Phi$ in $\mathbb{D}$, subject to the functor laws. The intuition is that $F$ defines sets of elements in various $\mathbb{D}$-contexts, together with a $\mathbb{D}$-substitution action. A morphism $f\colon F \to G$ is a natural transformation, which is a family of functions $f_\Psi\colon F(\Psi) \to G(\Psi)$ for any $\Psi$. This family of functions must be natural, i.e. commute with substitution $f_\Psi \circ F(\sigma) = F(\sigma) \circ f_\Phi$.

For the purposes of modelling higher-order abstract syntax, $\mathbb{D}$ will typically be the term model of some domain-level lambda-calculus. By domain-level, we mean the calculus that serves as the meta-level for object-language encodings. It is the calculus that contains constants like `lam` and `app` from the Introduction. We call it domain-level to avoid possible confusion between different meta-levels later. For simplicity, let us for now use a simply-typed lambda-calculus with functions and products as the domain language. It is sufficient to encode the example from the Introduction and allows us to explain the main idea underlying our approach.

The term model of the simply-typed domain-level lambda-calculus forms a cartesian closed category $\mathbb{D}$. The objects of $\mathbb{D}$ are contexts $x_1{:}\,A_1, \dots, x_n{:}\,A_n$ of simple types. We use $\Phi$ and $\Psi$ to range over such contexts. A morphism from $x_1{:}\,A_1, \dots, x_n{:}\,A_n$ to $x_1{:}\,B_1, \dots, x_m{:}\,B_m$ is a tuple $(t_1, \dots, t_m)$ of terms $x_1{:}\,A_1, \dots, x_n{:}\,A_n \vdash t_i\colon B_i$ for $i = 1, \dots, m$. A morphism of type $\Psi \to \Phi$ in $\mathbb{D}$ thus amounts to a (domain-level) substitution that provides a (domain-level) term in context $\Psi$ for each of the variables in $\Phi$. Terms are identified up to $\alpha\beta\eta$-equality. One may achieve this by using a de Bruijn encoding, for example, but the specific encoding is not important for this paper. The terminal object is the empty context, which we denote by 1, and the product $\Phi \times \Psi$ is defined by context concatenation. It is not hard to see that any object $x_1{:}\,A_1, \dots, x_n{:}\,A_n$ is isomorphic to an object that is given by a context with a single variable, namely $x_1{:}\,(A_1 \times \dots \times A_n)$. This is to say that contexts can be identified with product types. In view of this isomorphism, we shall allow ourselves to consider the objects of $\mathbb{D}$ also as types and vice versa. The category $\mathbb{D}$ is cartesian closed, the exponential of $\Phi$ and $\Psi$ being given by the function type $\Phi \to \Psi$ (where the objects are considered as types).

The presheaf category $\widehat{\mathbb{D}}$ is a computational universe that both embeds the term model $\mathbb{D}$ and that can represent computations about it. Note that we cannot just enrich $\mathbb{D}$ with terms for computations if we want to use HOAS. In a simply-typed lambda-calculus with just the constant terms $\texttt{app}\colon \texttt{tm} \to \texttt{tm} \to \texttt{tm}$ and $\texttt{lam}\colon (\texttt{tm} \to \texttt{tm}) \to \texttt{tm}$, each term of type `tm` represents an object-level term. This would not be the true anymore, if we were to allow computations in the domain language, since one could define `M` to be something like ($\lambda$x. **if** x represents an object-level application **then** M1 **else** M2) for distinct `M1` and `M2`. In this case, `lam M` would not represent an object-level term anymore. If we want to preserve a bijection between the object-level terms and their representations in the domain-language, we cannot allow case-distinction over whether a term represents an object-level an application.

The category $\widehat{\mathbb{D}}$ unites syntax with computations by allowing one to enforce various degrees of substitution-invariance. By choosing objects with different substitution actions, one can control the required amount of substitution-invariance.

In one extreme, a set $S$ can be represented by the constant presheaf $\Delta S$ with $\Delta S(\Psi) = S$ and $\Delta S(\sigma) = \mathsf{id}$ for all $\Psi$ and $\sigma$. The substitution action is trivial. As a consequence, a morphism $\Delta S \to \Delta T$ amounts to a function from set $S$ to set $T$, since the trivial choice of the substitution action makes the naturality condition vacuous.

The Yoneda embedding represents the other extreme. For any object $\Phi$ of $\mathbb{D}$, the presheaf $\mathrm{y}(\Phi)\colon \mathbb{D}^{\mathrm{op}} \to \mathrm{Set}$ is defined by $\mathrm{y}(\Phi)(\Psi) = \mathbb{D}(\Psi, \Phi)$, which is the set of morphisms from $\Psi$ to $\Phi$ in $\mathbb{D}$. The functor action is pre-composition. The presheaf $\mathrm{y}(\Phi)$ should be understood as the type of all domain-level substitutions with codomain $\Phi$. An important example is $\texttt{Tm} := y(\texttt{tm})$. In this case, $\texttt{Tm}(\Psi)$ is the set of all morphisms of type $\Psi \to \texttt{tm}$ in $\mathbb{D}$. By the definition of $\mathbb{D}$, these correspond to domain-level terms of type `tm` in context $\Psi$. In this way, the presheaf `Tm` represents the domain-level terms of type `tm`.

The Yoneda embedding does in fact embed $\mathbb{D}$ into $\widehat{\mathbb{D}}$ fully and faithfully. The Yoneda embedding becomes a functor $\mathrm{y}\colon \mathbb{D} \to \widehat{\mathbb{D}}$ if one defines the morphism action to be post-composition. This means that y maps a morphism $\sigma\colon \Psi \to \Phi$ in $\mathbb{D}$ to the natural transformation $\mathrm{y}(\sigma)\colon \mathrm{y}(\Psi) \to \mathrm{y}(\Phi)$ that is defined by post-composing with $\sigma$. This definition makes y into a functor $\mathrm{y}\colon \mathbb{D} \to \widehat{\mathbb{D}}$ that is moreover full and faithful: its action on morphisms is a bijection from $\mathbb{D}(\Psi, \Phi)$ to $\widehat{\mathbb{D}}(\mathrm{y}(\Psi), \mathrm{y}(\Phi))$ for any $\Psi$ and $\Phi$. This is because a natural transformation $f\colon \mathrm{y}(\Psi) \to \mathrm{y}(\Phi)$ is, by naturality, uniquely determined by $f_\Psi(\mathsf{id})$, where $\mathsf{id} \in \mathbb{D}(\Psi, \Psi) = \mathrm{y}(\Psi)(\Psi)$, and $f_\Psi(\mathsf{id})$ is an element of $y(\Phi)(\Psi) = \mathbb{D}(\Psi, \Phi)$.

Since $\mathbb{D}$ embeds into $\widehat{\mathbb{D}}$ fully and faithfully, the term model of the domain language is available in $\widehat{\mathbb{D}}$. Consider for example $\texttt{Tm} = \mathrm{y}(\texttt{tm})$. Since y is full and faithful, the morphisms from `Tm` to `Tm` in $\widehat{\mathbb{D}}$ are in one-to-one correspondence with the morphisms from `tm` to `tm` in $\mathbb{D}$. These, in turn, are defined to be substitutions and correspond to simply-typed (domain-level) lambda terms with one free variable. This shows that substitution invariance cuts down the morphisms from `Tm` to `Tm` in $\widehat{\mathbb{D}}$ just as much as one would like for HOAS encodings.

But $\widehat{\mathbb{D}}$ contains not just a term model of the domain language. It can also represent computations about the domain-level syntax and computations that are not substitution-invariant. For example, arbitrary functions on terms can be represented as morphisms from the constant presheaf $\Delta(\texttt{Tm}(1))$ to `Tm`. Recall that 1

is the empty context, so that $\mathtt{Tm}(1)$ is the set $\mathbb{D}(1, \mathtt{tm})$, by definition, which is isomorphic to the set of closed domain-level terms of type $\mathtt{tm}$. The morphisms from $\Delta(\mathtt{Tm}(1))$ to $\mathtt{Tm}$ in $\widehat{\mathbb{D}}$ correspond to arbitrary functions from closed terms to closed terms, without any restriction of substitution invariance.

The restriction to the constant presheaf of closed terms can be generalised to arbitrary presheaves. Define a functor $\flat\colon \widehat{\mathbb{D}} \to \widehat{\mathbb{D}}$ by letting $\flat F$ be the constant presheaf $\Delta(F(1))$, i.e. $\flat F(\Psi) = F(1)$ and $\flat F(\sigma) = \mathsf{id}$. Thus, $\flat$ restricts any presheaf to the set of its closed elements. The functor $\flat$ defines a comonad where the counit $\varepsilon_F\colon \flat F \to F$ is the obvious inclusion and the comultiplication $\nu_F\colon \flat F \to \flat\flat F$ is the identity. The latter means that the comonad $\flat$ is idempotent.

### 3 Internal Language

To explain how $\widehat{\mathbb{D}}$ models higher-order abstract syntax and contextual types, we need to expose more of its structure. Most of this structure is standard. Defining it directly in terms of functors and natural transformations is somewhat laborious and the technical details may obscure the basic idea of our approach.

We therefore use the internal type theory of $\widehat{\mathbb{D}}$ as a meta-language for working with its structure. The structure of $\widehat{\mathbb{D}}$ furnishes a model of a dependent type theory that supports dependent products, dependent sums and extensional identity types, among others, in a standard way [11]. We use Agda notation for the types and terms of this internal type theory. We write $(x\colon S) \to T$ for a dependent function type and write $\lambda x\colon S.m$ and $m\ n$ for the associated lambda-abstractions and applications. As usual, we will sometimes also write $S \to T$ for $(x\colon S) \to T$ if $x$ does not appear in $T$. However, to make it easier to distinguish the function spaces at various levels, we will write $(x\colon S) \to T$ by default even when $x$ does not appear in $T$. We use $\mathtt{let}\ x = m\ \mathtt{in}\ n$ as an abbreviation for $(\lambda x\colon T.n)\ m$, as usual. For two terms $m\colon T$ and $n\colon T$, we write $m =_T n$ or just $m = n$ for the associated identity type. Our notation is similar to Agda's, since the internal type theory can be seen as a fragment of Agda's type theory. Agda has been useful as a tool for type-checking our constructions in the internal type theory [1].

In the spirit of Martin-Löf type theory, we will define basic types and terms successively as they are needed. In the Agda development this corresponds to postulating constants that are justified by the interpretation in $\widehat{\mathbb{D}}$. In the following sections, we will expose the structure of $\widehat{\mathbb{D}}$ step by step until we have enough to interpret contextual types.

While much of the structure of $\widehat{\mathbb{D}}$ can be captured by adding rules and constants to standard Martin-Löf type theory, for the comonad $\flat$ such a formulation would not be very satisfactory. The issues are discussed by Shulman [27, p.7], for example. To obtain a more satisfactory syntax for the comonad, we refine the internal type theory into a modal type theory in which $\flat$ appears as a necessity modality. This approach goes back to [3,4,6] and is also used by recent work of Shulman [27], Licata et al. [16] and others on working with the $\flat$-modality in type theory. Agda has recently gained support for such a $\flat$-modality [29].

We summarise here the typing rules for the $\flat$-modality which we will rely on. To control the modality, one uses two kinds of variables. In addition to standard variables $x{:}T$, one has a second kind of so-called *crisp* variables $x{::}T$. Typing judgements have the form $\Delta \mid \Theta \vdash m{:}T$, where $\Delta$ collects the crisp variables and $\Theta$ collects the ordinary variables. In essence, a crisp variable $x{::}T$ represents an assumption of the form $x{:}\flat T$. The syntactic distinction is useful, since it leads to a type theory that is well-behaved with respect to substitution, see [6,27].

The typing rules are closely related to those in modal type systems [6,18], where $\Delta$ is the typing context for modal (global) assumptions and $\Theta$ for (local) assumptions, and type systems for linear logic [4], where $\Delta$ is the typing context for non-linear assumptions and $\Theta$ for linear assumptions.

$$\frac{}{\Delta, u{::}T, \Delta' \mid \Theta \vdash u{:}T} \qquad \frac{}{\Delta \mid \Theta, x{:}T, \Theta' \vdash x{:}T}$$

$$\frac{\Delta \mid \cdot \vdash m : T}{\Delta \mid \Theta \vdash \texttt{box}\ m : \flat T} \qquad \frac{\Delta \mid \Theta \vdash m : \flat T \quad \Delta, x{::}T \mid \Theta \vdash n : S}{\Delta \mid \Theta \vdash \texttt{let box}\ x = m\ \texttt{in}\ n : S}$$

Given any term $m{:}\ T$ which only depends on modal variable context $\Delta$, we can form the term $\texttt{box}\ m{:}\ \flat T$. We have a let-term $\texttt{let box}\ x = m\ \texttt{in}\ n$ that takes a term $m{:}\ \flat T$ and binds it to a variable $x{::}T$. The rules maintain the invariant that the free variables in a type $\flat T$ or a term $\texttt{box}\ m$ are all crisp variables from the crisp context $\Delta$.

The other typing rules do not modify the crisp context. For examples, the rules for dependent products are:

$$\frac{\Delta \mid \Theta, x{:}T \vdash m{:}S}{\Delta \mid \Theta \vdash \lambda x{:}T.m : (x{:}T) \to S} \qquad \frac{\Delta \mid \Theta \vdash m{:}(y{:}T) \to S \quad \Delta \mid \Theta \vdash n{:}T}{\Delta \mid \Theta \vdash m\ n{:}[n/y]S}$$

When $\Delta$ is empty, we shall write just $\Theta \vdash m{:}T$ for $\Delta \mid \Theta \vdash m{:}T$.

### 4 From Presheaves to Contextual Types

Armed with the internal type theory, we can now explore the structure of $\widehat{\mathbb{D}}$.

#### 4.1 A Universe of Representables

For our purposes, the main feature of $\widehat{\mathbb{D}}$ is that it embeds $\mathbb{D}$ fully and faithfully via the Yoneda embedding. In the type theory for $\widehat{\mathbb{D}}$, we may capture this embedding by means of a Tarski-style universe. Such a universe is defined by a type of codes for types together with a decoding function that maps codes to actual types.

The type of codes $\texttt{Obj}$ represents the set of objects of $\mathbb{D}$ in the internal type theory of $\widehat{\mathbb{D}}$. We have seen above that any set can be represented as a presheaf with trivial substitution action, and $\texttt{Obj}$ is one such example. Particular objects of $\mathbb{D}$ then appear as terms of type $\texttt{Obj}$. The cartesian closed structure of $\mathbb{D}$ gives us terms $\texttt{unit}$, $\texttt{times}$, $\texttt{arrow}$ for the terminal object 1, finite products $\times$ and the exponential (function type). We also have a term for the domain-level type $\texttt{tm}$.

$$\vdash \texttt{Obj}\ \text{type} \qquad \vdash \texttt{tm}{:}\ \texttt{Obj} \qquad \vdash \texttt{times}{:}\ (a{:}\texttt{Obj}) \to (b{:}\texttt{Obj}) \to \texttt{Obj}$$
$$\vdash \texttt{unit}{:}\ \texttt{Obj} \qquad \vdash \texttt{arrow}{:}\ (a{:}\texttt{Obj}) \to (b{:}\texttt{Obj}) \to \texttt{Obj}$$

Subsequently, we sometimes talk about objects of $\mathbb{D}$ when we intend to describe terms of type $\texttt{Obj}$ (and vice versa).

The morphisms of $\mathbb{D}$ could similarly be encoded as a constant presheaf with many term constants, but this is in fact not necessary. Instead, we can use the Yoneda embedding as a function that decodes elements of $\texttt{Obj}$ into actual types.

$$x\colon \texttt{Obj} \vdash \texttt{El}\, x \text{ type}$$

The function $\texttt{El}$ is almost direct syntax for the Yoneda embedding. The interpretation in $\widehat{\mathbb{D}}$ is such that, for any object $A$ of $\mathbb{D}$, the type $\texttt{El}\, A$ is interpreted by the presheaf $\mathrm{y}(A)$. Such a presheaf is called *representable*. One can think of $\texttt{El}\, A$ as the type of all morphisms of type $\Psi \to A$ in $\mathbb{D}$ for arbitrary $\Psi$. Recall from above that a morphism of type $\Psi \to A$ in $\mathbb{D}$ amounts to a domain-level term of type $A$ that may refer to variables in $\Psi$. In this sense, one should think of $\texttt{El}\, A$ as a type of domain-level terms of type $A$, both closed and open ones.

We get all morphisms of $\mathbb{D}$, and no more, in this way, since the Yoneda embedding is full and faithful, recall Sec. 2. In our case, this means that the type $(x\colon \texttt{El}\, A) \to \texttt{El}\, B$ represents the morphisms of type $A \to B$ in $\mathbb{D}$. Any closed term of type $(x : \texttt{El}\, A) \to \texttt{El}\, B$ corresponds to such a morphism and vice versa. This is because the naturality requirements in $\widehat{\mathbb{D}}$ enforce substitution-invariance, as outlined in Sec. 2. The type $(x : \texttt{El}\, A) \to \texttt{El}\, B$ thus does not represent arbitrary functions from terms of type $A$ to terms of type $B$, but only substitution-invariant ones. If a function of this type maps a domain-level variable $x\colon A$ (encoded as an element of $\texttt{El}\, A$) to some term $M\colon B$ (encoded as an element of $\texttt{El}\, B$), then it must map any other $N\colon A$ to $[N/x]M$.

We note that the type dependency in $\texttt{El}$ is easy to work with. A term of type $(a\colon \texttt{Obj}) \to (b\colon \texttt{Obj}) \to (x\colon \texttt{El}\, a) \to \texttt{El}\, b$ corresponds to a family of terms $(x\colon \texttt{El}\, A) \to \texttt{El}\, B$ indexed by objects $A$ and $B$ in $\mathbb{D}$. This is because $\texttt{Obj}$ is just a set, so that the naturality constraints of $\widehat{\mathbb{D}}$ are vacuous for functions out of $\texttt{Obj}$.

To summarise, we get access to $\mathbb{D}$ in the internal type theory of $\widehat{\mathbb{D}}$ simply by considering the Yoneda embedding as the decoding function $\texttt{El}$ of a universe á la Tarski. Since is consists of the representable presheaves, we call it the *universe of representables*. The following lemmas state that the embedding preserves terminal object, binary products and the exponential.

**Lemma 1.** *The internal type theory of* $\widehat{\mathbb{D}}$ *has a term* $\vdash \texttt{terminal}\colon \texttt{El}\, \texttt{unit}$, *such that* $x = \texttt{terminal}$ *holds for any* $x\colon \texttt{El}\, \texttt{unit}$.

**Lemma 2.** *The internal type theory of* $\widehat{\mathbb{D}}$ *justifies the terms below, such that* $\texttt{fst}\ (\texttt{pair}\, x\ y) = x$, $\texttt{snd}\ (\texttt{pair}\, x\ y) = y$, $z = \texttt{pair}\ (\texttt{fst}\ z)\ (\texttt{snd}\ z)$ *for all* $x, y, z$.

$$c\colon \texttt{Obj},\ d\colon \texttt{Obj} \vdash \texttt{fst}\colon\ (z : \texttt{El}\, (\texttt{times}\ c\ d)) \to \texttt{El}\, c$$
$$c\colon \texttt{Obj},\ d\colon \texttt{Obj} \vdash \texttt{snd}\colon\ (z : \texttt{El}\, (\texttt{times}\ c\ d)) \to \texttt{El}\, d$$
$$c\colon \texttt{Obj},\ d\colon \texttt{Obj} \vdash \texttt{pair}\colon\ (x : \texttt{El}\, c) \to (y : \texttt{El}\, d) \to \texttt{El}\, (\texttt{times}\ c\ d)$$

**Lemma 3.** *The internal type theory of* $\widehat{\mathbb{D}}$ *justifies the terms below such that* $\texttt{arrow-i}\ (\texttt{arrow-e}\ f) = f$ *and* $\texttt{arrow-e}\ (\texttt{arrow-i}\ g) = g$ *for all* $f, g$.

$$c\colon \texttt{Obj},\ d\colon \texttt{Obj} \vdash \texttt{arrow-e}\colon\ (x\colon \texttt{El}\, (\texttt{arrow}\ c\ d)) \to (y\colon \texttt{El}\, c) \to \texttt{El}\, d$$
$$c\colon \texttt{Obj},\ d\colon \texttt{Obj} \vdash \texttt{arrow-i}\colon\ (y\colon (\texttt{El}\, c \to \texttt{El}\, d)) \to \texttt{El}\, (\texttt{arrow}\ c\ d)$$

#### 4.2 Higher-Order Abstract Syntax

The last lemma in the previous section states that $\mathtt{El}\,A \to \mathtt{El}\,B$ is isomorphic to $\mathtt{El}\,(\mathtt{arrow}\;A\;B)$. This is particularly useful to lift HOAS-encodings from $\mathbb{D}$ to $\widehat{\mathbb{D}}$. For instance, the domain-level term constant $\mathtt{lam}\colon (\mathtt{tm} \to \mathtt{tm}) \to \mathtt{tm}$ gives rise to an element of $\mathtt{El}\,(\mathtt{arrow}\;(\mathtt{arrow}\;\mathtt{tm}\;\mathtt{tm})\;\mathtt{tm})$. But this type is isomorphic to $(\mathtt{El}\,\mathtt{tm} \to \mathtt{El}\,\mathtt{tm}) \to \mathtt{El}\,\mathtt{tm}$, by the lemma.

This means that the higher-order abstract syntax constants lift to $\widehat{\mathbb{D}}$:

$$\mathtt{app}\colon (m\colon \mathtt{El}\,\mathtt{tm}) \to (n\colon \mathtt{El}\,\mathtt{tm}) \to \mathtt{El}\,\mathtt{tm} \qquad \mathtt{lam}\colon (m\colon (\mathtt{El}\,\mathtt{tm} \to \mathtt{El}\,\mathtt{tm})) \to \mathtt{El}\,\mathtt{tm}$$

Once one recognises $\mathtt{El}\,A$ as $\mathrm{y}(A)$, the adequacy of this higher-order abstract syntax encoding lifts from $\mathbb{D}$ to $\widehat{\mathbb{D}}$ as in [12]. For example, an argument $M$ to $\mathtt{lam}$ has type $\mathtt{El}\,\mathtt{tm} \to \mathtt{El}\,\mathtt{tm}$, which is isomorphic to $\mathtt{El}\,(\mathtt{arrow}\;\mathtt{tm}\;\mathtt{tm})$. But this type represents (open) domain-level terms $t\colon \mathtt{tm} \to \mathtt{tm}$. The term $\mathtt{lam}\;M\colon \mathtt{El}\,\mathtt{tm}$ then represents the domain-level term $\mathtt{lam}\;t\colon \mathtt{tm}$, so it just lifts the domain-level.

#### 4.3 Closed Objects

One should think of $\flat T$ as the type of 'closed' elements of $T$. In particular, $\flat(\mathtt{El}\,A)$ represents morphisms of type $1 \to A$ in $\mathbb{D}$, recall the definition of $\flat$ from Sec. 2 and that $\mathtt{El}\,A$ corresponds to $\mathrm{y}A$. In the term model $\mathbb{D}$, the morphisms $1 \to A$ correspond to closed domain-language terms of type $A$. Thus, while $\mathtt{El}\,A$ represents both open and closed domain-level terms, $\flat(\mathtt{El}\,A)$ represents only the closed ones.

This applies also to the type $\mathtt{El}\,A \to \mathtt{El}\,B$. We have seen above that $\mathtt{El}\,A \to \mathtt{El}\,B$ is isomorphic to $\mathtt{El}\,(\mathtt{arrow}\;A\;B)$ and may therefore be thought of as containing the terms of type $B$ with a distinguished variable of type $A$. But, these terms may contain other free domain language variables. The type $\flat(\mathtt{El}\,A \to \mathtt{El}\,B)$, on the other hand, contains only terms of type $B$ that may contain (at most) one variable of type $A$.

Restricting to closed object with the modality is useful because it disables substitution-invariance. For example, the internal type theory for $\widehat{\mathbb{D}}$ justifies a function $\mathtt{is\text{-}lam}\colon (x{:}\flat(\mathtt{El}\,\mathtt{tm})) \to \mathtt{bool}$ that returns $\mathtt{true}$ if and only if the argument represents a domain language lambda abstraction. We shall define it in the next section. Such a function cannot be defined with type $\mathtt{El}\,\mathtt{tm} \to \mathtt{bool}$, since it would not be invariant under substitution. Its argument ranges over terms that may be open; which particularly includes domain-level variables. The function would have to return $\mathtt{false}$ for them, since a domain-level variable is not a lambda-abstraction. But after substituting a lambda-abstraction for the variable, it would have to return $\mathtt{true}$, so it could not be substitution-invariant.

We note that the type $\mathtt{Obj}$ consists only of closed elements and that $\mathtt{Obj}$ and $\flat\mathtt{Obj}$ happen to be definitionally equal types (an isomorphism would suffice, but equality is more convenient).

#### 4.4 Contextual Objects

Using function types and the modality, it is now possible to work with contextual objects that represent domain level terms in a certain context, much like in [20,21]. A contextual type $\lceil \Psi \vdash A \rceil$ is a boxed function type of the form $\flat(\mathtt{El}\,\Psi \to \mathtt{El}\,A)$. It represents domain-level terms of type $A$ with variables from $\Psi$. Here, we consider the domain-level context $\Psi$ as a term that encodes it. The interpretation will make this precise.

For example, domain-level terms with up to two free variables now appear as terms of type $\flat(\mathtt{El}\,((\mathtt{times}\,(\mathtt{times}\,\mathtt{unit}\,\mathtt{tm})\,\mathtt{tm}) \to \mathtt{El}\,\mathtt{tm})$, as the following example illustrates.

$$\begin{aligned} \mathtt{box}\,(\lambda u{:}\,\mathtt{El}\,((\mathtt{times}\,(\mathtt{times}\,\mathtt{unit}\,\mathtt{tm})\,\mathtt{tm}).\ & \mathtt{let}\ x_1 = \mathtt{snd}\,(\mathtt{fst}\,u)\ \mathtt{in} \\ & \mathtt{let}\ x_2 = \mathtt{snd}\,u\ \mathtt{in} \\ & \quad \mathtt{app}\,(\mathtt{lam}\,(\lambda x{:}\,\mathtt{El}\,\mathtt{tm}.\,\mathtt{app}\,x_1\,x))\,x_2\ ) \end{aligned}$$

The context variables $x_1$ and $x_2$ are bound at the meta level.

This representation integrates substitution as usual. For example, given crisp variables $m{::}\mathtt{El}\,(\mathtt{times}\,c\,\mathtt{tm}) \to \mathtt{tm}$ and $n{::}\mathtt{El}\,c \to \mathtt{tm}$ for contextual terms, the term $\mathtt{box}\,(\lambda u{:}\,\mathtt{El}\,c.\,m\,(\mathtt{pair}\,u\,(n\,u)))$ represents substitution of $n$ for the last variable in the context of $m$.

For working with contextual objects, it is convenient to lift the constants app and lam to contextual types.

$$\begin{aligned} c{:}\,\mathtt{Obj} &\vdash \mathtt{app}'{:}\ \flat(\mathtt{El}\,c \to \mathtt{El}\,\mathtt{tm}) \to \flat(\mathtt{El}\,c \to \mathtt{El}\,\mathtt{tm}) \to \flat(\mathtt{El}\,c \to \mathtt{tm}) \\ c{:}\mathtt{Obj} &\vdash \mathtt{lam}'{:}\ \flat(\mathtt{El}\,(\mathtt{times}\,c\,\mathtt{tm}) \to \mathtt{El}\,\mathtt{tm}) \to \flat(\mathtt{El}\,c \to \mathtt{El}\,\mathtt{tm}) \end{aligned}$$

These terms are defined by:

$$\begin{aligned} \mathtt{app}' &:= \lambda m, n.\,\mathtt{let}\ \mathtt{box}\ m' = m\ \mathtt{in}\ \mathtt{let}\ \mathtt{box}\ n' = n\ \mathtt{in} \\ &\qquad\qquad \mathtt{box}\,(\lambda u{:}\,\mathtt{El}\,c.\,\mathtt{app}\,(m'\,u)\,(n'\,u)) \\ \mathtt{lam}' &:= \lambda m.\,\mathtt{let}\ \mathtt{box}\ m' = m\ \mathtt{in}\ \mathtt{box}\,(\lambda u{:}\,\mathtt{El}\,c.\,\mathtt{lam}\,(\lambda x{:}\,\mathtt{El}\,\mathtt{tm}.\ m'\,(\mathtt{pair}\,u\,x))) \end{aligned}$$

A contextual type for domain-level variables (as opposed to arbitrary terms) can be defined by restricting the function space in $\flat(\mathtt{El}\,\Psi \to \mathtt{El}\,A)$ to consist only of projections. Projections are functions of the form $\mathtt{snd} \circ \mathtt{fst}_k$, where we write $\mathtt{fst}_k$ for the $k$-fold iteration $\mathtt{fst} \circ \cdots \circ \mathtt{fst}$. Let us write $S \to_v T$ for the subtype of $S \to T$ consisting only of projections. The contextual type $\flat(\mathtt{El}\,\Psi \to_v \mathtt{El}\,A)$ is then a subtype of $\flat(\mathtt{El}\,\Psi \to \mathtt{El}\,A)$.

With these definitions, we can express a primitive recursion scheme for contextual types. We write it in its general form where the result type $A$ can possibly depend on $x$. This is only relevant for the dependently typed case; in the simply typed case, the only dependency is on $c$.

**Lemma 4.** *Let $c{:}\,\mathtt{Obj}$, $x{:}\,\flat(\mathtt{El}\,c \to \mathtt{El}\,\mathtt{tm}) \vdash A\ c\ x$ type and define:*

$$\begin{aligned} X_{var} &:= (c{:}\,\mathtt{Obj}) \to (x{:}\,\flat(\mathtt{El}\,c \to_v \mathtt{El}\,\mathtt{tm})) \to A\ c\ x \\ X_{app} &:= (c{:}\,\mathtt{Obj}) \to (x, y{:}\,\flat(\mathtt{El}\,c \to \mathtt{El}\,\mathtt{tm})) \to A\ c\ x \to A\ c\ y \to A\ c\ (\mathtt{app}'\ x\ \ y) \\ X_{lam} &:= (c{:}\,\mathtt{Obj}) \to (x{:}\,\flat(\mathtt{El}\,(\mathtt{times}\ c\ \mathtt{tm}) \to \mathtt{El}\,\mathtt{tm})) \to A\ (\mathtt{times}\ c\ \mathtt{tm})\ x \to A\ c\ (\mathtt{lam}'\ x) \end{aligned}$$

*Then,* $\widehat{\mathbb{D}}$ *justifies a term*

$$\vdash \mathit{rec}\colon X_{\mathit{var}} \to X_{\mathit{app}} \to X_{\mathit{lam}} \to (c\colon \mathit{Obj}) \to (x\colon \flat(\mathit{El}\, c \to \mathit{El}\; \mathit{tm})) \to A\; c\; x$$

*such that the following equations are valid.*

$$\begin{aligned}
&\mathit{rec}\, t_{\mathit{var}}\, t_{\mathit{app}}\, t_{\mathit{lam}}\, c\; x &&= t_{\mathit{var}}\; c\; x && \text{if } x\colon \flat(\mathit{El}\, c \to_v \mathit{El}\; \mathit{tm})\\
&\mathit{rec}\, t_{\mathit{var}}\, t_{\mathit{app}}\, t_{\mathit{lam}}\, c\; (\mathit{app}'\; s\; t) &&= t_{\mathit{app}}\; c\; s\; t\\
&\mathit{rec}\, t_{\mathit{var}}\, t_{\mathit{app}}\, t_{\mathit{lam}}\, c\; (\mathit{lam}'\; s) &&= t_{\mathit{lam}}\; c\; s
\end{aligned}$$

*Proof (outline).* To outline the proof idea, note first that a function of type $(c\colon \mathtt{Obj}) \to (x\colon \flat(\mathtt{El}\, c \to \mathtt{El}\,\mathtt{tm})) \to A\; c\; x$ in $\widehat{\mathbb{D}}$, corresponds to an inhabitant of $A\; \Phi\; t$ for each concrete object $\Phi$ of $\mathbb{D}$ and each inhabitant $t\colon \flat(\mathtt{El}\,\Phi \to \mathtt{El}\,\mathtt{tm})$. This is because naturality constraints for boxed types are vacuous (and $\mathtt{Obj} = \flat\mathtt{Obj}$). Next, note that inhabitants of $\flat(\mathtt{El}\,\Phi \to \mathtt{El}\,\mathtt{tm})$ correspond to domain-level terms of type $\mathtt{tm}$ in context $\Phi$ up to $\alpha\beta\eta$-equality. We can perform a case-distinction on whether it is a variable, abstraction or application and depending on the result use $t_{\mathtt{var}}$, $t_{\mathtt{app}}$ or $t_{\mathtt{lam}}$ to define the required inhabitant of $A\; \Phi\; t$.

As a simple example for `rec`, we can define the function `is-lam` discussed above by $\mathtt{rec}\ (\curlywedge c, x.\, \mathtt{false})\ (\curlywedge c, x, y, r_x, r_y.\, \mathtt{false})\ (\curlywedge c, x, r_x.\, \mathtt{true})$.

### 5 Simple Contextual Modal Type Theory

We have outlined informally how the internal dependent type theory of $\widehat{\mathbb{D}}$ can model contextual types. In this section, we make this precise by giving the interpretation of Cocon [23], a contextual modal type theory where we can work with contextual HOAS trees and computations about them, into $\widehat{\mathbb{D}}$. We will focus here on a simply-typed version of Cocon where we use a simply-typed domain-language with constants `app` and `lam` and also only allow computations about HOAS trees, but do not consider, for example, universes. Concentrating on a stripped down, simply-typed version of Cocon allows us to focus on the essential aspects, namely how to interpret domain-level contexts and domain-level contextual objects and types semantically. The generalisation to a dependently typed domain-level such as LF in Sec. 6 will be conceptually straightforward, although more technical. Handling universes is an orthogonal issue (see also [16]).

We first define our simply-typed domain-level with the type `tm` the term constants `lam` and `app` (see Fig. 1). Following Cocon, we allow computations to be embedded into domain-level terms via unboxing. The intuition is that if a program $t$ promises to compute a value of type $\lceil x{:}\mathtt{tm}, y{:}\mathtt{tm} \vdash \mathtt{tm}\rceil$, then we can embed $t$ directly into a domain-level object writing $\mathtt{lam}\ \lambda x.\mathtt{lam}\ \lambda y.\mathtt{app}\ \lfloor t \rfloor\ x$, unboxing $t$. Domain-level objects (resp. types) can be packaged together with their domain-level context to form a contextual object (resp. type). Domain-level contexts are formed as usual, but may contain context variables to describe a yet unknown prefix. Last, we include domain-level substitutions that allow us to move between domain-level contexts. The compound substitution $\sigma, M$ extends the substitution $\sigma$ with domain $\widehat{\Psi}$ to a substitution with domain $\widehat{\Psi}, x$, where $M$ replaces $x$.

| | | | |
|---|---|---|---|
| Domain-level types | $A, B$ | $::=$ | $\mathtt{tm} \mid A \to B$ |
| Domain-level terms | $M, N$ | $::=$ | $\lambda x.M \mid M\,N \mid x \mid \mathsf{lam} \mid \mathsf{app} \mid \lfloor t \rfloor_\sigma$ |
| Domain-level contexts | $\Psi, \Phi$ | $::=$ | $\cdot \mid \psi \mid \Psi, x{:}A$ |
| Domain-level context (erased) | $\widehat{\Psi}, \widehat{\Phi}$ | $::=$ | $\cdot \mid \psi \mid \widehat{\Psi}, x$ |
| Domain-level substitutions | $\sigma$ | $::=$ | $\cdot \mid \mathsf{wk}_{\widehat{\Psi}} \mid \sigma, M$ |
| Contextual types | $T$ | $::=$ | $\Psi \vdash A \mid \Psi \vdash_{\overline{v}} A$ |
| Contextual objects | $C$ | $::=$ | $\widehat{\Psi} \vdash M$ |
| Domain of discourse | $\breve{\tau}$ | $::=$ | $\tau \mid \mathtt{ctx}$ |
| Types and Terms | $\tau, \mathcal{I}$ | $::=$ | $\lceil T \rceil \mid (y : \breve{\tau}_1) \Rightarrow \tau_2$ |
| | $t, s$ | $::=$ | $y \mid \lceil C \rceil \mid \mathsf{rec}^{\mathcal{I}}\ \boldsymbol{\mathcal{B}}\ \Psi\ t \mid \mathsf{fn}\ y \Rightarrow t \mid t_1\ t_2$ |
| Branches | $\mathcal{B}$ | $::=$ | $\Gamma \mapsto t$ |
| Contexts | $\Gamma$ | $::=$ | $\cdot \mid \Gamma, y : \breve{\tau}$ |

**Fig. 1.** Syntax of COCON with a fixed simply-typed domain $\mathtt{tm}$

Following [18,23], we do not store the domain (like $\widehat{\Psi}$) in the substitution, it can always be recovered before applying the substitution. We also include *weakening substitution*, written as $\mathsf{wk}_{\widehat{\Psi}}$, to describe the weakening of the domain $\Psi$ to $\Psi, \overrightarrow{x{:}A}$. Weakening substitutions are necessary, as they allow us to express the weakening of a context variable $\psi$. Identity is a special form of the $\mathsf{wk}_{\widehat{\Psi}}$ substitution, which follows immediately from the typing rule of $\mathsf{wk}_{\widehat{\Psi}}$. Composition is admissible.

We summarise the typing rules for domain-level terms and types in Fig. 2. We also include typing rules for domain-level contexts. Note that since we restrict ourselves to a simply-typed domain-level, we simply check that $A$ is a well-formed type. We defer the reduction and expansion rules to the appendix and only remark here that equality for domain-level terms and substitution is modulo $\beta\eta$. In particular, $\lfloor \lceil \widehat{\Phi} \vdash N \rceil \rfloor_\sigma$ reduces to $[\sigma]N$.

In our grammar, we distinguish between the contextual type $\Psi \vdash A$ and the more restricted contextual type $\Phi \vdash_{\overline{v}} A$ which characterises only variables of type $A$ from the domain-level context $\Phi$. We give here two sample typing rules for $\Phi \vdash_{\overline{v}} A$ which are the ones used most in practice to illustrate the main idea. We embed contextual objects into computations via the modality. Computation-level types include boxed contextual types, $\lceil \Phi \vdash A \rceil$, and function types, written as $(y : \breve{\tau}_1) \Rightarrow \tau_2$. We overload the function space and allow as domain of discourse both computation-level types and the schema $\mathsf{ctx}$ of domain-level context, although only in the latter case $y$ can occur in $\tau_2$. We use $\mathsf{fn}\ y \Rightarrow t$ to introduce functions of both kinds. We also overload function application $t\ \ s$ to eliminate function types $(y : \tau_1) \Rightarrow \tau_2$ and $(y : \mathsf{ctx}) \Rightarrow \tau_2$, although in the latter case $s$ stands for a domain-level context. We separate domain-level contexts from contextual objects, as we do not allow functions that return a domain-level context.

The recursor is written as $\mathsf{rec}^{\mathcal{I}}\ \boldsymbol{\mathcal{B}}\ \Psi\ t$. Here, $t$ describes a term of type $\lceil \Psi \vdash \mathtt{tm} \rceil$ that we recurse over and $\boldsymbol{\mathcal{B}}$ describes the different branches that we can take

$\boxed{\Gamma; \Psi \vdash M : A}$ Term $M$ has type $A$ in domain-level context $\Psi$ and context $\Gamma$

$$\frac{\Gamma \vdash \Psi : \mathtt{ctx} \quad x{:}A \in \Psi}{\Gamma; \Psi \vdash x : A} \qquad \frac{\Gamma \vdash \Psi : \mathtt{ctx}}{\Gamma; \Psi \vdash \mathsf{lam} : (\mathtt{tm} \to \mathtt{tm}) \to \mathtt{tm}} \qquad \frac{\Gamma \vdash \Psi : \mathtt{ctx}}{\Gamma; \Psi \vdash \mathsf{app} : \mathtt{tm} \to \mathtt{tm} \to \mathtt{tm}}$$

$$\frac{\Gamma; \Psi \vdash M : A \to B \quad \Gamma; \Psi \vdash N : A}{\Gamma; \Psi \vdash M\ N : B} \qquad \frac{\Gamma; \Psi, x{:}A \vdash M : B}{\Gamma; \Psi \vdash \lambda x.M : A \to B}$$

$$\frac{\Gamma \vdash t : \lceil \Phi \vdash A \rceil \quad \Gamma; \Psi \vdash \sigma : \Phi}{\Gamma; \Psi \vdash \lfloor t \rfloor_\sigma : A}$$

$\boxed{\Gamma; \Phi \vdash \sigma : \Psi}$ Substitution $\sigma$ provides a mapping from the (domain) context $\Psi$ to $\Phi$

$$\frac{\Gamma \vdash \Psi, \overrightarrow{x{:}A} : \mathtt{ctx}}{\Gamma; \Psi, \overrightarrow{x{:}A} \vdash \mathsf{wk}_{\widehat{\Psi}} : \Psi} \qquad \frac{\Gamma \vdash \Phi : \mathtt{ctx}}{\Gamma; \Phi \vdash \cdot : \cdot} \qquad \frac{\Gamma; \Phi \vdash \sigma : \Psi \quad \Gamma; \Phi \vdash M : A}{\Gamma; \Phi \vdash \sigma, M : \Psi, x{:}A}$$

$\boxed{\Gamma \vdash \Psi : \mathtt{ctx}}$ Domain-level context $\Psi$ is a well-formed

$$\frac{}{\Gamma \vdash \cdot : \mathtt{ctx}} \qquad \frac{\Gamma(y) = \mathsf{ctx}}{\Gamma \vdash y : \mathtt{ctx}} \qquad \frac{\Gamma \vdash \Psi : \mathtt{ctx}}{\Gamma \vdash \Psi, x{:}A : \mathtt{ctx}}$$

**Fig. 2.** Typing Rules for Domain-level Terms, Substitutions, Contexts depending on the value computed by $t$. As is common when we have dependencies, we annotate the recursor with the typing invariant $\mathcal{I}$. Here, we consider only the recursor over domain-level terms of type $\mathtt{tm}$. Hence, we annotate it with $\mathcal{I} = (\psi : \mathsf{ctx}) \Rightarrow (y : \lceil \psi \vdash \mathtt{tm} \rceil) \Rightarrow \tau$. To check that the recursor $\mathsf{rec}^{\mathcal{I}}\ \mathcal{B}\ \Psi\ t$ has type $[\Psi/\psi]\tau$, we check that each of the three branches has the specified type $\mathcal{I}$. In the base case, we may assume in addition to $\psi : \mathsf{ctx}$ that we have a variable $p : \lceil \psi \vdash_v \mathtt{tm} \rceil$ and check that the body has the appropriate type. If we encounter a contextual object built with the domain-level constant $\mathsf{app}$, then we choose the branch $b_{\mathsf{app}}$. We assume $\psi{:}\,\mathsf{ctx}$, $m{:}\,\lceil \psi \vdash \mathtt{tm} \rceil$, $n{:}\,\lceil \psi \vdash \mathtt{tm} \rceil$, as well as $f_n$ and $f_m$ which stand for the recursive calls on $m$ and $n$ respectively. We then check that the body $t_{\mathsf{app}}$ is well-typed. If we encounter a domain object built with the domain-level constant $\mathsf{lam}$, then we choose the branch $b_{\mathsf{lam}}$. We assume $\psi{:}\,\mathsf{ctx}$ and $m{:}\,\lceil \psi, x{:}\mathtt{tm} \vdash \mathtt{tm} \rceil$ together with the recursive call $f_m$ on $m$ in the extended LF context $\psi, x{:}\mathtt{tm}$. We then check that the body $t_{\mathsf{lam}}$ is well-typed. The typing rules for computations are given in Fig. 3. We omit the reduction rules here and refer the interested reader to the appendix.

#### 5.1 Interpretation

We now give an interpretation of simply-typed Cocon in a presheaf model with a cartesian closed universe of representables. Let us first extend the internal dependent type theory with the constant $\mathtt{tm}$ for modelling the domain-level type constant $\mathtt{tm}$ and with the constants $\mathtt{app}\colon \mathtt{El}\,\mathtt{tm} \to \mathtt{El}\,\mathtt{tm} \to \mathtt{El}\,\mathtt{tm}$ and $\mathtt{lam}\colon (\mathtt{El}\,\mathtt{tm} \to$

$\boxed{\Gamma \vdash C : T}$ Contextual object $C$ has contextual type $T$

$$\frac{\Gamma; \Psi \vdash M : A}{\Gamma \vdash (\widehat{\Psi} \vdash M) : (\Psi \vdash A)} \qquad \frac{\Gamma \vdash \Psi : \mathtt{ctx} \quad x{:}A \in \Psi}{\Gamma \vdash (\widehat{\Psi} \vdash x) : (\Psi \vdash_v A)} \qquad \frac{x{:}\lceil \Phi \vdash_v A \rceil \in \Gamma \quad \Gamma; \Psi \vdash \mathsf{wk}_{\widehat{\Psi}} : \Phi}{\Gamma \vdash (\widehat{\Psi} \vdash \lfloor x \rfloor_{\mathsf{wk}_{\widehat{\Psi}}}) : (\Psi \vdash_v A)}$$

$\boxed{\Gamma \vdash t : \tau}$ Term $t$ has computation type $\tau$

$$\frac{y : \check{\tau} \in \Gamma}{\Gamma \vdash y : \check{\tau}} \qquad \frac{\Gamma \vdash C : T}{\Gamma \vdash \lceil C \rceil : \lceil T \rceil}$$

$$\frac{\Gamma \vdash t : (y : \check{\tau}_1) \Rightarrow \tau_2 \quad \Gamma \vdash s : \check{\tau}_1}{\Gamma \vdash t\ s : [s/y]\tau_2} \qquad \frac{\Gamma, y : \check{\tau}_1 \vdash t : \tau_2 \quad \Gamma \vdash (y : \check{\tau}_1) \Rightarrow \tau_2 : \text{ type}}{\Gamma \vdash \mathsf{fn}\ y \Rightarrow t : (y : \check{\tau}_1) \Rightarrow \tau_2}$$

Recursor over domain-level terms $\mathcal{I} = (\psi : \mathsf{ctx}) \Rightarrow (y : \lceil \psi \vdash \mathtt{tm} \rceil) \Rightarrow \tau$

$$\frac{\Gamma \vdash t : \lceil \Psi \vdash \mathtt{tm} \rceil \quad \Gamma \vdash \mathcal{I} : u \quad \Gamma \vdash b_v : \mathcal{I} \quad \Gamma \vdash b_{\mathsf{app}} : \mathcal{I} \quad \Gamma \vdash b_{\mathsf{lam}} : \mathcal{I}}{\Gamma \vdash \mathsf{rec}^{\mathcal{I}}(b_v \mid b_{\mathsf{app}} \mid b_{\mathsf{lam}})\ \ \Psi\ t : [\Psi/\psi]\tau}$$

Branch for Variable $$\frac{\Gamma, \psi : \mathsf{ctx}, p : \lceil \psi \vdash_v \mathtt{tm} \rceil \vdash t_v : \tau}{\Gamma \vdash (\psi, p \mapsto t_v) : \mathcal{I}}$$

Branch for Application $\mathsf{app}$ $$\frac{\Gamma, \psi : \mathsf{ctx}, m{:}\lceil \psi \vdash \mathtt{tm} \rceil, n{:}\lceil \psi \vdash \mathtt{tm} \rceil, f_m{:}\tau, f_n{:}\tau \vdash t_{\mathsf{app}} : \tau}{\Gamma \vdash (\psi, m, n, f_n, f_m \mapsto t_{\mathsf{app}}) : \mathcal{I}}$$

Branch for Function $\mathsf{lam}$ $$\frac{\Gamma, \phi : \mathsf{ctx}, m{:}\lceil \phi, x{:}\mathtt{tm} \vdash \mathtt{tm} \rceil, f_m{:}[(\phi, x{:}\mathtt{tm})/\psi]\tau \vdash t_{\mathsf{lam}} : [\phi/\psi]\tau}{\Gamma \vdash \psi, m, f_m \mapsto t_{\mathsf{lam}} : \mathcal{I}}$$

**Fig. 3.** Typing Rules for Contextual Objects and Computations

$\mathtt{El}\,\mathtt{tm}) \to \mathtt{El}\,\mathtt{tm}$ to model the corresponding domain-level constants $\mathsf{app}$ and $\mathsf{lam}$.

We can now translate domain-level and computation-level types of Cocon into the internal dependent type theory for $\widehat{\mathbb{D}}$. We do so by interpreting the domain-level terms, types, substitutions, and contexts (see Fig. 4). All translations are on well-typed terms and types. Domain-level types are interpreted as the terms of type $\mathtt{Obj}$ in the internal dependent type theory that represent them. Domain-level contexts are also interpreted as terms of type $\mathtt{Obj}$ by $[\![\Gamma \vdash \Psi : \mathtt{ctx}]\!]$. For example, a domain-level context $x{:}\mathtt{tm}, y{:}\mathtt{tm}$ is interpreted as $\mathtt{times}\ (\mathtt{times}\ \mathtt{unit}\ \mathtt{tm})\ \mathtt{tm} : \mathtt{Obj}$. A domain-level substitution with domain $\Psi$ and codomain $\Phi$ becomes a term of type $\mathtt{El}\, e'$ that is parameterised by an element $u{:}\,\mathtt{El}\, e$, where $e = [\![\Gamma \vdash \Phi : \mathtt{ctx}]\!]$ and $e' = [\![\Gamma \vdash \Psi : \mathtt{ctx}]\!]$. As $e'$ is some product, for example $\mathtt{times}\ (\mathtt{times}\ \mathtt{unit}\ \mathtt{tm})\ \mathtt{tm}$, the domain-level substitution is translated into an n-ary tuple. A weakening substitution $\Gamma; \Psi, x{:}\mathtt{tm} \vdash \mathsf{wk}_{\Psi} : \Psi$ is interpreted as $\mathtt{fst}\ u$ where $u{:}\,\mathtt{El}\ (\mathtt{times}\ e\ \mathtt{tm})$ and $e = [\![\Gamma \vdash \Psi : \mathtt{ctx}]\!]$. More generally, when we weaken a context $\Psi$ by $n$ declarations, i.e. $\overrightarrow{x{:}A}$, we interpret $\mathsf{wk}_{\Psi}$ as $\mathtt{fst}_n\ u$.

A well-typed domain-level term, $\Gamma; \Psi \vdash M : A$, is mapped to an object of type $\mathtt{El}\,[\![A]\!]$ that depends on $u{:}\mathtt{El}\,[\![\Gamma \vdash \Psi : \mathtt{ctx}]\!]$.

Hence the translation of a well-typed domain-level term is indexed by $u$ that stands for the term-level interpretation of a domain-level context $\Phi$. Initially, $u$

Interpretation of domain-level types

$$[\![\mathtt{tm}]\!] = \mathtt{tm}$$
$$[\![A \to B]\!] = \mathtt{arrow}\ [\![A]\!]\ [\![B]\!]$$

Interpretation of domain-level contexts

$$[\![\Gamma \vdash \psi : \mathtt{ctx}]\!] = \psi$$
$$[\![\Gamma \vdash \cdot : \mathtt{ctx}]\!] = \mathtt{unit}$$
$$[\![\Gamma \vdash (\Psi, x{:}A) : \mathtt{ctx}]\!] = \mathtt{times}\ e\ [\![A]\!] \qquad \text{where } [\![\Gamma \vdash \Psi : \mathtt{ctx}]\!] = e$$

Interpretation of domain-level terms where $u{:}\,\mathtt{El}\,e$ and $[\![\Gamma \vdash \Psi : \mathtt{ctx}]\!] = e$

$$[\![\Gamma; \Psi \vdash x{:}\ A]\!]_u = \mathtt{snd}\ (\mathtt{fst}_k\ u) \qquad \text{where } \Psi = \Psi_0, x{:}A, y_k{:}A_k, \ldots, y_1{:}A_1$$
$$[\![\Gamma; \Psi \vdash \lambda x.\, M : A \to B]\!]_u = \mathtt{arrow\text{-}i}\ (\curlywedge x{:}\mathtt{El}\ [\![A]\!].\ e) \qquad \text{where } [\![\Gamma; \Psi, x{:}A \vdash M : B]\!]_{(\mathtt{pair}\ u\ x)} = e$$
$$[\![\Gamma; \Psi \vdash M\ N : B]\!]_u = \mathtt{arrow\text{-}e}\ e_1\ e_2 \qquad \text{where } [\![\Gamma; \Psi \vdash M : A \to B]\!]_u = e_1 \text{ and } [\![\Gamma; \Psi \vdash N : A]\!]_u = e_2$$
$$[\![\Gamma; \Psi \vdash \lfloor t \rfloor_\sigma : A]\!]_u = \mathtt{let\ box}\ x = e_1\ \mathtt{in}\ x\ e_2 \qquad \text{where } [\![\Gamma \vdash t : \lceil \Phi \vdash A \rceil]\!] = e_1 \text{ and } [\![\Gamma; \Psi \vdash \sigma : \Phi]\!]_u = e_2$$
$$[\![\Gamma; \Psi \vdash \mathsf{app} : \mathtt{tm} \to \mathtt{tm} \to \mathtt{tm}]\!]_u = \mathtt{arrow\text{-}i}(\curlywedge x{:}\mathtt{El}\,\mathtt{tm}.\ \mathtt{arrow\text{-}i}\ (\curlywedge y{:}\mathtt{El}\,\mathtt{tm}.\ \mathtt{app}\ x\ y))$$
$$[\![\Gamma; \Psi \vdash \mathsf{lam} : (\mathtt{tm} \to \mathtt{tm}) \to \mathtt{tm}]\!]_u = \mathtt{arrow\text{-}i}(\curlywedge f{:}\mathtt{El}\,(\mathtt{arrow}\ \mathtt{tm}\ \mathtt{tm}).\ \mathtt{lam}\ (\curlywedge x{:}\mathtt{El}\,\mathtt{tm}.\ \mathtt{arrow\text{-}e}\ f\ x))$$

Interpretation of domain-level substitutions where $u{:}\,\mathtt{El}\ e$ and $[\![\Gamma \vdash \Phi : \mathtt{ctx}]\!] = e$

$$[\![\Gamma; \Psi \vdash \cdot : \cdot]\!]_u = \mathtt{terminal}$$
$$[\![\Gamma; \Psi \vdash (\sigma, M) : \Phi, x{:}A]\!]_u = \mathtt{pair}\ e_1\ e_2 \qquad \text{where } [\![\Gamma; \Psi \vdash \sigma : \Phi]\!]_u = e_1 \text{ and } [\![\Gamma; \Psi \vdash M : A]\!]_u = e_2$$
$$[\![\Gamma; \Psi, \overrightarrow{x{:}A} \vdash \mathsf{wk}_{\widehat{\Phi}} : \Phi]\!]_u = \mathtt{fst}_n\ u \qquad \text{where } n = |\overrightarrow{x{:}A}|$$

**Fig. 4.** Interpretation of Domain-level Types and Terms is simply a variable. However, when we translate $\Gamma; \Phi \vdash \lambda x.M : A \to B$ given $u{:}\,\mathtt{El}\,e$ where $[\![\Gamma \vdash \Psi : \mathtt{ctx}]\!] = e$, we need to recursively translate $M$ in the extended domain-level context $\Psi, x{:}A$ and hence we also need to build a term $\mathtt{pair}\ u\ x$ that inhabits $\mathtt{El}\,(\mathtt{times}\ e\ [\![A]\!])$. The translation of $\Gamma; \Phi, x{:}A \vdash M : A$ will return a term $e$ that may contain $x$. However, note that $x$ will eventually be bound in $\mathtt{arrow\text{-}i}\ (\curlywedge x{:}\mathtt{El}\ [\![A]\!].\ e)$ When we translate a variable $x$ where $\Phi = \Phi_0, x{:}A, y_k{:}A_k, \ldots, y_1{:}A_1$, we return $\mathtt{fst}_k\ (\mathtt{snd}\ u)$. We translate $\Gamma; \Phi \vdash \lfloor t \rfloor_\sigma : A$ directly using $\mathtt{let\ box}$-construct where the domain-level substitution $\sigma$ is simply translated into a pair. As the computation $t$ has the contextual type $\lceil \Psi \vdash \mathtt{tm} \rceil$ its translation will be of type $\flat(\mathtt{El}\,e \to \mathtt{El}\,\mathtt{tm})$ where $e = [\![\Gamma \vdash \Psi : \mathtt{ctx}]\!]$. Hence we simply can extract a function $x{:}(\mathtt{El}\,e \to \mathtt{El}\,\mathtt{tm})$ using $\mathtt{let\ box}$ construct and pass to it the interpretation of $\sigma$. The translation of domain-level applications and domain-level constants $\mathsf{app}$ and $\mathsf{lam}$ is straightforward.

The interpretation of a contextual types $\lceil \Psi \vdash A \rceil$ makes explicit the fact that they correspond to functions $\mathtt{El}\,e \to \mathtt{El}\,[\![A]\!]$ where $e = [\![\Gamma \vdash \Psi : \mathtt{ctx}]\!]$ (see Fig. 5). Consequently, the corresponding contextual object $(\widehat{\Phi} \vdash M)$ is interpreted as a

Interpretation of contextual objects ($C$)

$$[\![\Gamma \vdash (\widehat{\Phi} \vdash M) : (\Phi \vdash A)]\!] = \lambda u{:}\, \mathtt{El}\, e.\, e' \quad \text{where } [\![\Gamma \vdash \Phi : \mathtt{ctx}]\!] = e \text{ and } [\![\Gamma ; \Phi \vdash M : A]\!]_u = e'$$

$$[\![\Gamma \vdash (\widehat{\Phi} \vdash M) : (\Phi \vdash_v A)]\!] = \lambda u{:}\, \mathtt{El}\, e.\, e' \quad \text{where } [\![\Gamma \vdash \Phi : \mathtt{ctx}]\!] = e \text{ and } [\![\Gamma ; \Phi \vdash M : A]\!]_u = e'$$

Interpretation of contextual types ($T$)

$$[\![\Gamma \vdash (\Phi \vdash A)]\!] = (u{:}\mathtt{El}\, e) \to \mathtt{El}\, [\![A]\!] \quad \text{where } [\![\Gamma \vdash \Phi : \mathtt{ctx}]\!] = e$$

$$[\![\Gamma \vdash (\Phi \vdash_v A)]\!] = (u{:}\mathtt{El}\, e) \to_v \mathtt{El}\, [\![A]\!] \quad \text{where } [\![\Gamma \vdash \Phi : \mathtt{ctx}]\!] = e$$

**Fig. 5.** Interpretation of Contextual Objects and Types

Interpretation of computation-level types ($\breve{\tau}$)

$$[\![\lceil T \rceil]\!] = \flat [\![T]\!]$$

$$[\![(x{:}\breve{\tau}_1) \Rightarrow \tau_2]\!] = (x{:}[\![\breve{\tau}_1]\!]) \to [\![\tau_2]\!]$$

$$[\![\mathtt{ctx}]\!] = \mathtt{Obj}$$

Computation-level typing contexts ($\Gamma$)

$$[\![\cdot]\!] = \cdot$$

$$[\![\Gamma,\, x{:}\, \breve{\tau}]\!] = [\![\Gamma]\!],\, x{:}\, [\![\breve{\tau}]\!]$$

Interpretation of computations ($\Gamma \vdash t : \tau$; without recursor)

$$[\![\Gamma \vdash \lceil C \rceil : \lceil T \rceil]\!] = \mathtt{box}\ e \quad \text{where } [\![\Gamma \vdash C : T]\!] = e$$

$$[\![\Gamma \vdash t_1\ t_2 : \tau]\!] = e_1\ e_2 \quad \text{where } [\![\Gamma \vdash t_1 : (x{:}\breve{\tau}_2) \Rightarrow \tau]\!] = e_1 \text{ and } [\![\Gamma \vdash t_2 : \breve{\tau}_2]\!] = e_2$$

$$[\![\Gamma \vdash \mathsf{fn}\ x \Rightarrow t : (x{:}\breve{\tau}_1) \Rightarrow \tau_2]\!] = \lambda x{:}\, [\![\breve{\tau}_1]\!].\, e \quad \text{where } [\![\Gamma, x{:}\breve{\tau}_1 \vdash t : \tau_2]\!] = e$$

$$[\![\Gamma \vdash x : \tau]\!] = x$$

**Fig. 6.** Interpretation of Computation-level Types and Terms – without recursor function. Similarly, $\lceil \Psi \vdash_v A \rceil$ is mapped to the restricted function space denoted by $\to_v$, which describes functions with bodies that only contain projections.

Last, we give the interpretation of computation-level types, contexts and terms (see Fig. 6). It is mostly straightforward, as we simply map $\lceil T \rceil$ to $\flat [\![T]\!]$ and $\lceil C \rceil$ is simply interpreted as boxed term.

The interpretation of the recursor is also straightforward now (see Fig. 7). In Lemma 4, we expressed a primitive recursion scheme in our internal type theory and defined a term `rec` together with its type. We now interpret every branch of our recursor in the computation-level as a function of the required type in our internal type theory. While this is somewhat tedious, it is straightforward.

We can now show that all well-typed domain-level and computation-level objects are translated into well-typed constructions in our internal type theory. As a consequence, we can show that equality in Cocon is equivalent to the corresponding equivalence in our internal type theoretic interpretation.

Interpretation of recursor for $\mathcal{I} = (\psi : \mathsf{ctx}) \Rightarrow (y : \lceil \psi \vdash \mathtt{tm} \rceil) \Rightarrow \tau$ :

$[\![\Gamma \vdash \mathsf{rec}^{\mathcal{I}}(b_v \mid b_{\mathsf{app}} \mid b_{\mathsf{lam}})\ \ \Psi\ t : [\Psi/\psi,\ t/y]\tau]\!] = \mathtt{rec}\ e_v\ e_{\mathsf{app}}\ e_{\mathtt{lam}}\ e_c\ e$
where $[\![\Gamma \vdash b_v : \mathcal{I}]\!] = e_v, [\![\Gamma \vdash b_{\mathsf{app}} : \mathcal{I}]\!] = e_{\mathsf{app}}, [\![\Gamma \vdash b_{\mathsf{lam}} : \mathcal{I}]\!] = e_{\mathtt{lam}}$,
$[\![\Gamma \vdash \Psi : \mathtt{ctx}]\!] = e_c$ and $[\![\Gamma \vdash t : \lceil \Psi \vdash \mathtt{tm} \rceil]\!] = e$

Interpretation of Variable Branch

$[\![\Gamma \vdash (\psi, x \mapsto t_v) : \mathcal{I}]\!] \qquad = \curlywedge\psi{:}\,\mathtt{Obj}.\ \curlywedge x{:}\,\flat(\mathtt{El}\,\psi \to_v \mathtt{El}\,\mathtt{tm}).\,e$
where $[\![\Gamma, \psi : \mathsf{ctx}, x : \lceil \psi \vdash_v \mathtt{tm} \rceil \vdash t_v : [x/y]\tau]\!] = e$

Interpretation of Application Branch

$[\![\Gamma \vdash (\psi, m, n, f_n, f_m \mapsto t_{\mathsf{app}}) : \mathcal{I}]\!] = \curlywedge\psi{:}\,\mathtt{Obj}.\ \curlywedge m, n{:}\,\flat(\mathtt{El}\,\psi \to \mathtt{El}\,\mathtt{tm}).$
$\curlywedge f_m{:}\,[\![[m/y]\tau]\!].\ \curlywedge f_n{:}\,[\![[n/y]\tau]\!].\,e$
where $[\![\Gamma, \psi{:}\mathtt{ctx}, m{:}\lceil \psi \vdash \mathtt{tm} \rceil, n{:}\lceil \psi \vdash \mathtt{tm} \rceil \vdash t_{\mathsf{app}} : [\lceil \psi \vdash \mathsf{app}\ \lfloor m \rfloor\ \lfloor n \rfloor \rceil / y]\tau]\!] = e$

Interpretation of Lambda-Abstraction Branch

$[\![\Gamma \vdash (\psi, m, f_m \mapsto t_{\mathsf{lam}}) : \mathcal{I}]\!] \qquad = \curlywedge\psi{:}\,\mathtt{Obj}.\curlywedge m{:}\,\flat(\mathtt{El}\,(\mathtt{times}\ \psi\ \mathtt{tm}) \to \mathtt{El}\,\mathtt{tm}).$
$\curlywedge f_m{:}\tau_m.e$
where $[\![[(\psi, x{:}\mathtt{tm})/\psi,\ m/y]\tau]\!] = \tau_m$,
$[\![\Gamma, \psi{:}\mathtt{ctx}, m{:}\lceil \psi, x{:}\mathtt{tm} \vdash \mathtt{tm} \rceil \vdash t_{\mathsf{app}} : [\lceil \psi \vdash \mathsf{lam}\ \lambda x.\lfloor m \rfloor \rceil / y]\tau]\!] = e$

**Fig. 7.** Interpretation of Recursor

**Lemma 5.** *The interpretation maintains the following typing invariants:*

- *If* $\Gamma \vdash \Psi{:}\ \mathtt{ctx}$ *then* $[\![\Gamma \vdash \Psi{:}\ \mathtt{ctx}]\!]{:}\ \mathtt{Obj}$.
- *If* $\Gamma; \Psi \vdash M{:}\ A$ *then* $[\![\Gamma]\!],\ u{:}\,\mathtt{El}\,[\![\Gamma \vdash \Psi : \mathtt{ctx}]\!] \vdash [\![\Gamma; \Psi \vdash M{:}\ A]\!]_u{:}\ \mathtt{El}\,[\![A]\!]$.
- *If* $\Gamma; \Psi \vdash \sigma{:}\ \Psi$ *then* $[\![\Gamma]\!],\ u{:}\,\mathtt{El}\,[\![\Gamma \vdash \Psi : \mathtt{ctx}]\!] \vdash [\![\Gamma; \Psi \vdash \sigma{:}\ \Psi]\!]_u{:}\ \mathtt{El}\,[\![\Psi]\!]$.
- *If* $\Gamma \vdash C{:}\ T$ *then* $[\![\Gamma]\!] \vdash [\![\Gamma \vdash C : T]\!]{:}\ [\![T]\!]$.
- *If* $\Gamma \vdash t{:}\ \tau$ *then* $[\![\Gamma]\!] \vdash [\![\Gamma \vdash t{:}\ \tau]\!]{:}\ [\![\tau]\!]$.

The proof goes by induction on derivations.

**Proposition 1 (Soundness).** *The following are true.*

- *If* $\Gamma; \Psi \vdash M \equiv N{:}\ A$ *then*
  $[\![\Gamma]\!],\ u{:}\,\mathtt{El}\,[\![\Psi]\!] \vdash [\![\Gamma;\ \Psi \vdash M : A]\!]_u = [\![\Gamma;\ \Psi \vdash N : A]\!]_u{:}\ \mathtt{El}\,[\![A]\!]$.
- *If* $\Gamma;\Psi \vdash \sigma \equiv \sigma'{:}\ \Phi$ *then*
  $[\![\Gamma]\!], u{:}\,\mathtt{El}\,[\![\Psi]\!] \vdash [\![\Gamma;\Psi \vdash \sigma : \Phi]\!]_u = [\![\Gamma;\Psi \vdash \sigma' : \Phi]\!]_u{:}\ \mathtt{El}\,[\![\Phi]\!]$.
- *If* $\Gamma \vdash t_1 \equiv t_2{:}\ \tau$ *then* $[\![\Gamma]\!] \vdash [\![\Gamma \vdash t_1 : \tau]\!] = [\![\Gamma \vdash t_2 : \tau]\!]{:}\ [\![\tau]\!]$.

### 6 Presheaves on a Small Category with Attributes

To explain the core of our approach as simply as possible, we have concentrated on a simply-typed domain language. In the remaining space, we outline how our approach generalises to dependent domain languages like LF.

We follow the same approach as above. We start from a term model $\mathbb{D}$ of the domain language and then interpret contextual types in the presheaf category $\widehat{\mathbb{D}}$. In the simply-typed case above, $\mathbb{D}$ was a small cartesian closed category. In the dependent case, $\mathbb{D}$ is a small *Category with Attributes*. Categories with attributes (CwAs) [11] are a general notion of model for dependent type theories that is suitable for modelling dependent domain languages like LF.

With this change, we follow essentially the same approach as above. The main difference is that the universe of representables now makes available the CwA-structure of $\mathbb{D}$ instead of the cartesian closed structure. The following section outlines this in analogy to Sec. 4.1.

#### 6.1 Yoneda CwA

In a Yoneda CwA we again have a type for the objects of $\mathbb{D}$, which we now denote $\mathtt{Ctx}$. In the term model for LF, these would be the LF contexts. The type $\mathtt{Ty}\ c$ represents (possibly dependent) LF types in context $c$. Contexts can be built with the constants $\mathtt{nil}$ and $\mathtt{cons}$.

$$\begin{array}{rl} \vdash \mathtt{Ctx}\ \text{type} & \qquad \vdash \mathtt{nil}\colon \mathtt{Ctx} \\ c\colon \mathtt{Ctx} \vdash \mathtt{Ty}\ c\ \text{type} & \qquad \vdash \mathtt{cons}\colon (c\colon \mathtt{Ctx}) \to (a\colon \mathtt{Ty}\ c) \to \mathtt{Ctx} \end{array}$$

Both $\mathtt{Ctx}$ and $\mathtt{Ty}\ c$ are constant presheaves, i.e. $\flat\mathtt{Ctx} = \mathtt{Ctx}$ and $\flat(\mathtt{Ty}\ c) = \mathtt{Ty}\ c$.

As in Sec. 4.1, we consider the contexts as codes of a universe.

$$c\colon \mathtt{Ctx} \vdash \mathtt{El}\, c\ \text{type}$$

The type $\mathtt{El}\, c$ has the same interpretation as before and is essentially just the Yoneda embedding. The morphisms $c \to d$ of the CwA $\mathbb{D}$ thus appear as functions of type $\mathtt{El}\, c \to \mathtt{El}\, d$.

The axioms of a CwA can be stated using terms and equations in the internal language of $\widehat{\mathbb{D}}$. For example, substitution on types and context projection morphisms are given by the following constants.

$$\begin{array}{r} c, d\colon \mathtt{Ctx} \vdash \mathtt{sub}\colon (a\colon \mathtt{Ty}\ d) \to (f\colon \mathtt{El}\, c \to \mathtt{El}\, d) \to \mathtt{Ty}\ c \\ c\colon \mathtt{Ctx},\ a\colon \mathtt{Ty}\ c \vdash p\colon \mathtt{El}\ (\mathtt{cons}\ c\ a) \to \mathtt{El}\, c \qquad\qquad \end{array}$$

The other components of a CwA are added similarly and the CwA-axioms [11] are expressed in terms of equations for these constants.

The inhabitants of a type can then be captured by the dependent type

$$c\colon \mathtt{Ctx},\ a\colon \mathtt{Ty}\ c,\ u\colon \mathtt{El}\, c \vdash \mathtt{I}\ a\ u\ \text{type}$$

defined by $\mathtt{I}\ a\ u\ :=\ \Sigma v\colon \mathtt{El}\,(\mathtt{cons}\ c\ a).\,(p\ v) = u$. This type contains all values in $\mathtt{El}\,(\mathtt{cons}\ c\ a)$ whose first projection is $u$. If one considers $u\colon \mathtt{El}\, c$ as a dependent tuple of LF terms (one term for each variable in the context represented by $c$), then $\mathtt{I}\ a\ u$ represents all the terms that can be appended to this tuple to make it into one of type $\mathtt{El}\,(\mathtt{cons}\ c\ a)$. Indeed, one can define a pairing operation by $\mathtt{pair} := \lambda u.\, \lambda\langle v, p\rangle.\, v$.

$$c\colon \mathtt{Ctx},\ a\colon (\mathtt{Ty}\ c) \vdash \mathtt{pair}\colon (u\colon \mathtt{El}\, c) \to \mathtt{I}\ a\ u \to \mathtt{El}\,(\mathtt{cons}\ c\ a)$$

With these definitions, we can represent dependent contextual types much like the simply-typed ones. Recall that we had interpreted $\Phi \vdash A$ by $\mathtt{El}\,[\![\Phi]\!] \to \mathtt{El}\,[\![A]\!]$ where both $[\![\Phi]\!]$ and $[\![A]\!]$ were terms of type $\mathtt{Obj}$. In the dependent case, $A$ may depend on $\Phi$. The interpretation of $\Phi$ is a term $[\![\Phi]\!] : \mathtt{Ctx}$, much as before. The interpretation of $A$ takes the dependency into account: $u{:}\,\mathtt{El}\,[\![\Phi]\!] \vdash [\![A]\!]_u : \mathtt{Ty}\ u$. The interpretation of the contextual type $\Phi \vdash A$ will then be:

$$(u{:}\,\mathtt{El}\,[\![\Phi]\!]) \to \mathtt{I}\ [\![A]\!]_u\ u$$

It may be interesting to note that $(u{:}\,\mathtt{El}\,c) \to \mathtt{I}\ a\ u$ is isomorphic to the type of sections of $p\colon \mathtt{El}\,(\mathtt{cons}\ c\ a) \to \mathtt{El}\,c$.

Object-level term constants in LF can be lifted using $\mathtt{I}$. Consider, for example, an encoding of the simply-typed lambda-calculus in LF. It represents only well-typed terms by means of the constants $\mathtt{app}\colon \Pi a, b{:}\,\mathtt{ty}.\ \mathtt{tm}\,(\mathtt{arr}\ a\ b) \to \mathtt{tm}\ a \to \mathtt{tm}\ b$ and $\mathtt{lam}\colon \Pi a, b{:}\,\mathtt{ty}.\ (\mathtt{tm}\ a \to \mathtt{tm}\ b) \to \mathtt{tm}\,(\mathtt{arr}\ a\ b)$. Therein, the type $\mathtt{tm}$ of object-level terms is dependent on an object-level type $\mathtt{ty}$, which may be built using a constant $\mathtt{o}\colon \mathtt{ty}$ for a base type and a constant $\mathtt{arr}\colon \mathtt{ty} \to \mathtt{ty} \to \mathtt{ty}$ for function types. This encoding lifts to the Yoneda CwA as in simply-typed case:

$$\begin{array}{ll}
c{:}\,\mathtt{Ctx} \vdash \mathtt{ty}\colon \mathtt{Ty}\ c & \Gamma \vdash \mathtt{o}\colon \mathtt{I}\ \mathtt{ty}\ u \\
c{:}\,\mathtt{Ctx} \vdash \mathtt{tm}\colon \mathtt{Ty}\ (\mathtt{cons}\ c\ \mathtt{ty}) & \Gamma \vdash \mathtt{arr}\colon \mathtt{I}\ \mathtt{ty}\ u \to \mathtt{I}\ \mathtt{ty}\ u \to \mathtt{I}\ \mathtt{ty}\ u
\end{array}$$

$$\Delta \vdash \mathtt{app}\colon \mathtt{I}\ \mathtt{tm}\ (\mathtt{pair}\ u\ (\mathtt{arr}\ a\ b)) \to \mathtt{I}\ \mathtt{tm}\ (\mathtt{pair}\ u\ a) \to \mathtt{I}\ \mathtt{tm}\ (\mathtt{pair}\ u\ b)$$
$$\vdash \mathtt{lam}\colon\ (\mathtt{I}\ \mathtt{tm}\ (\mathtt{pair}\ u\ a) \to \mathtt{I}\ \mathtt{tm}\ (\mathtt{pair}\ u\ b)) \to \mathtt{I}\ \mathtt{tm}\ (\mathtt{pair}\ u\ (\mathtt{arr}\ a\ b))$$

Here, $\Gamma$ abbreviates $c{:}\,\mathtt{Ctx},\ u{:}\,(\mathtt{El}\,c)$ and $\Delta$ abbreviates $\Gamma,\ a, b{:}\,(\mathtt{I}\ \mathtt{ty}\ u)$. Notice how $\mathtt{lam}$ uses higher-order abstract syntax at the meta level.

With these definitions, the interpretation of Cocon is essentially just as before. For working with the dependencies in a Yoneda CwA, we found it very useful to type-check our definitions in Agda, see our sources [1].

## 7 Conclusion

We have given a rational reconstruction of contextual type theory in presheaf models of higher-order abstract syntax. This provides a semantical way of understanding the invariants of contextual types independently of the algorithmic details of type checking. At the same time, we identify the contextual modal type theory, Cocon, which is known to be normalising, as a syntax for presheaf models of HOAS. By accounting for the Yoneda embedding with a universe á la Tarski, we obtain a manageable way of constructing contextual types in the model, especially in the dependent case. While various forms of universes are being studied in the context of functor categories, e.g. [2,16], we are not aware of previous uses of presheaves over CwAs or similar.

In future work, one may consider using the model as a way of compiling contextual types, by implementing the semantics. In another direction, it may be interesting to apply the syntax of contextual types to other presheaf categories. We also hope that the model will help to guide the further development of Cocon.

*Acknowledgements.* We thank the anonymous reviewers for helpful feedback.

## References

[1] The Agda sources for this paper are available from: http://github.com/uelis/contextual.

[2] Guillaume Allais, Robert Atkey, James Chapman, Conor McBride, and James McKinna. A type and scope safe universe of syntaxes with binding: Their semantics and proofs. *Proc. ACM Program. Lang.*, 2(ICFP):90:1–90:30, July 2018.

[3] Benton, P.N., Bierman, G.M., de Paiva, V., Hyland, M.: A term calculus for intuitionistic linear logic. In: Bezem, M., Groote, J.F. (eds.) Typed Lambda Calculi and Applications, International Conference on Typed Lambda Calculi and Applications, TLCA ’93, Utrecht, The Netherlands, March 16-18, 1993, Proceedings. vol. 664, pp. 75–90. Springer (1993)

[4] Andrew Barber and Gordon Plotkin. Dual intuitionistic linear logic. Technical Report, LFCS, University of Edinburgh, 1997.

[5] John Cartmell. Generalised algebraic theories and contextual categories. *Annals of Pure and Applied Logic*, 32:209 – 243, 1986.

[6] Rowan Davies and Frank Pfenning. A modal analysis of staged computation. *Journal of the ACM*, 48(3):555–604, 2001.

[7] Peter Dybjer. Internal type theory. In *Types for Proofs and Programs (TYPES’95)*, pages 120–134, 1995.

[8] M. Fiore, G. D. Plotkin, and D. Turi. Abstract syntax and variable binding. In *Logic in Computer Science (LICS’99)*, pages 193–202. IEEE Press, 1999.

[9] Murdoch Gabbay and Andrew Pitts. A new approach to abstract syntax involving binders. In *Logic in Computer Science (LICS’99)*, pages 214–224. IEEE Press, 1999.

[10] Robert Harper, Furio Honsell, and Gordon Plotkin. A framework for defining logics. *Journal of the ACM*, 40(1):143–184, January 1993.

[11] Martin Hofmann. *Syntax and Semantics of Dependent Types*, page 79–130. Publications of the Newton Institute. Cambridge University Press, 1997.

[12] Martin Hofmann. Semantical analysis of higher-order abstract syntax. In *Logic in Computer Science (LICS’99)*, pages 204–213. IEEE Press, 1999.

[13] Furio Honsell, Marino Miculan, and Ivan Scagnetto. An axiomatic approach to metareasoning on nominal algebras in HOAS. In *International Colloquium on Automata, Languages and Programming (ICALP’01)*, LNCS 2076, pages 963–978. Springer, 2001.

[14] Bart Jacobs. Comprehension categories and the semantics of type dependency. *Theor. Comput. Sci.*, 107(2):169–207, 1993.

[15] Kavvos, G.A.: Intensionality, intensional recursion, and the Gödel-Löb axiom. CoRR **abs/1703.01288** (2017), http://arxiv.org/abs/1703.01288

[16] Daniel R. Licata, Ian Orton, Andrew M. Pitts, and Bas Spitters. Internal universes in models of homotopy type theory. In *Formal Structures for Computation and Deduction (FSCD’18)*, pages 22:1–22:17, 2018.

[17] Dale Miller and Catuscia Palamidessi. Foundational aspects of syntax. *ACM Comput. Surv.*, 31(3es), 1999.

[18] Aleksandar Nanevski, Frank Pfenning, and Brigitte Pientka. Contextual modal type theory. *ACM Transactions on Computational Logic*, 9(3):1–49, 2008.

[19] Frank Pfenning and Conal Elliott. Higher-order abstract syntax. In *Symposium on Language Design and Implementation (PLDI’88)*, pages 199–208, June 1988.

[20] Brigitte Pientka. A type-theoretic foundation for programming with higher-order abstract syntax and first-class substitutions. In *Principles of Programming Languages (POPL’08)*, pages 371–382. ACM Press, 2008.

[21] Brigitte Pientka and Andreas Abel. Well-founded recursion over contextual objects. In *Typed Lambda Calculi and Applications (TLCA’15)*, pages 273–287, 2015.

[22] Brigitte Pientka, Andreas Abel, Francisco Ferreira, David Thibodeau, and Rébecca Zucchini. Cocon: Computation in contextual type theory. *CoRR*, abs/1901.03378, 2019.

[23] Brigitte Pientka, Andreas Abel, Francisco Ferreira, David Thibodeau, and Rebecca Zucchini. A type theory for defining logics and proofs. In *34th IEEE/ ACM Symposium on Logic in Computer Science (LICS’19)*, pages 1–13, IEEE Computer Society, 2019.

[24] Brigitte Pientka and Andrew Cave. Inductive Beluga: Programming Proofs (System Description). In *Conference on Automated Deduction (CADE-25)*, LNCS 9195, pages 272–281. Springer, 2015.

[25] Brigitte Pientka and Joshua Dunfield. Programming with proofs and explicit contexts. In *Principles and Practice of Declarative Programming (PPDP’08)*, pages 163–173, 2008.

[26] Brigitte Pientka and Joshua Dunfield. Beluga: a framework for programming and reasoning with deductive systems (System Description). In *International Joint Conference on Automated Reasoning (IJCAR’10)*, LNAI 6173, pages 15–21. Springer, 2010.

[27] Shulman, M.: Brouwer’s fixed-point theorem in real-cohesive homotopy type theory. Mathematical Structures in Computer Science **28**(6), 856–941 (2018)

[28] Thomas Streicher. *Semantics of Type Theory*. Birkhäuser, 1991.

[29] Andrea Vezzosi. Agda with a flat modality. Available from https://github.com/agda/agda/tree/flat, 2018.

